# Anti-tumor Activity of N^4^ [(E)-1-(2-hydroxyphenyl) Methylidene], N^4^-[(E)-2-Phenylethylidene], N^4^ [(E,2E)-3-Phenyl-2-propenylidene], and N^4^ [(E)ethylidene] Isonicotinohydrazide on K562 and Jurkat Cell Lines

**DOI:** 10.4103/0975-1483.71638

**Published:** 2010

**Authors:** F Shabani, S Ghammamy, A Jahazi, F Siavoshifar

**Affiliations:** *Department of Chemistry, Islamic Azad University, Young Researchers Club, Ardabil, Iran*; 1 *Department of Chemistry, Islamic Azad University, Ardabil, Iran*; 2 *Moravvej Student-Research Institute, Ardabil Branch, Ardabil, Iran*; 3 *Department of Medicinal Sciences, Islamic Azad University, Ardabil, Iran*

**Keywords:** 4-pyridinecarboxylic acid hydrazide, isonicotinohydrazide, Jurkat, K562

## Abstract

Using the water eliminated mechanism, reactions of 4-pyridinecarboxylic acid hydrazide and salicylaldehyde, benzaldehyde, cinnamaldehyde, and formaldehyde afforded the corresponding N^4^[(E)-1-(2-hydroxyphenyl) methylidene] (NHPM), N^4^-[(E)-2-phenylethylidene] (NPI), N^4^[(E,2E)-3-phenyl-2-propenylidene] (NPPI), and N^4^[(E) ethylidene] (NEI) isonicotinohydrazide, in high yields, after several minutes, as reported. These new compounds have shown antitumor activity against two kinds of cancer cells, which are K562 (human chronic myeloid leukemia) and Jurkat (human T lymphocyte carcinoma).

## INTRODUCTION

Nitrogen-containing heterocyclic compounds are widespread in nature, and their applications to biologically active pharmaceuticals, agrochemicals, and functional materials are becoming more and more important.[1] Due to the existence of the pyridine ring in the structure of many biologically active compounds, for example herbicides such as, nicotinic acid, vitamin B_5_, nicotinamide vitamin B_6_, pyridonal and pyridonamine, and nicotinamide adenine dinucleotide, the pyridine ring has been studied extensively, both experimentally and theoretically.[2] The development of new efficient methods to synthesize N-heterocycles with structural diversity is one major interest of modern synthetic organic chemists.[3–5] Among a large variety of nitrogen-containing heterocyclic compounds, those containing bridgehead hydrazines have received considerable attention because of their pharmacological properties and clinical applications.[6,7] For example, 1-arylamino-2, 3-dihydro-1Hpyrazolo [1, 2-b] phthalazine-5,10-dione derivatives were reported to possess anti-inflammatory, analgesic, anti hypoxic, and antipyretic properties.[8]

From these points of view, it is interesting to study different types of biologically active ligands. In this article, the synthesis, characterization, and anti-tumor properties of a number of the new ligands have been studied.

## MATERIALS AND METHODS

4-pyridinecarboxylic acid hydrazide, salicylaldehyde, benzaldehyde, cinnamaldehyde, and formaldehyde were Merck chemicals, and were used without further purification. Organic solvents were of reagent grade. Electronic spectra were recorded by Camspec UV–Visible spectrophotometer model Wpa bio Wave S2 100. The IR spectra were recorded using the FT-IR Bruker Tensor 27 spectrometer.^1^HNMR was recorded on a Bruker AVANCE DRX 500 spectrometer. All the chemical shifts were quoted in ppm using the high-frequency positive convention;^1^H NMR spectra was referenced to external SiMe_4_. The percent composition of elements was obtained from the Microanalytical Laboratories, Department of Chemistry, OIRC, Tehran.

### Cell culture

The human chronic myeloid leukemia: K562 cell line and the human T lymphocyte carcinoma: Jurkat cell line, used for treatment with the drugs, was provided. K562 and Jurkat cells were grown at 37°C in an atmosphere containing 5% CO_2_, with RPMI-1640 MEDIUM HEPES Modification, with L-glutamine and 25 mM HEPES (SIGMA-ALDRICH CHEMIE GmbH), supplemented with 10% heat-inactivated fetal bovine serum (FBS) (Gibco), 2.7% sodium bicarbonate, and 500 mg/L ampicillin.

### Synthesis of the compounds; General method

**Figure 1:**
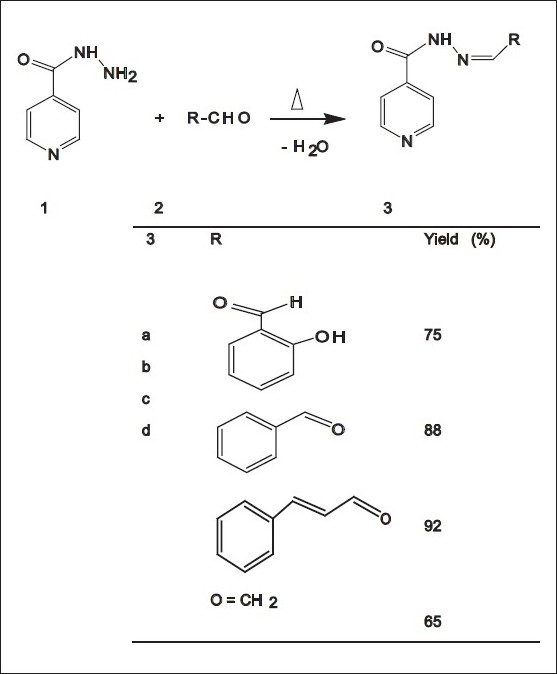
Synthesis route of compounds

To a magnetically stirred mixture of 4-Pyridinecarboxylic acid hydrazide (1.37 g, 10 mmol) in hot methanol (20 mL), one type of aldehyde, such as, salicylaldehyde (1.22 g,1 mmol), benzaldehyde (1.06 g, 1 mmol), cinnamaldehyde (1.39 g,1 mmol), or formaldehyde (0.321 g, 1 mmol) was added via a syringe and heated for 45 minutes at 60°C. After cooling to room temperature, the resulting precipitate was filtered and washed with hexane (20 mL) [Figure 1].

**Typical procedure for preparation of N^4^ [(E)-1-(2-hydroxyphenyl) methylidene] Isonicotinohydrazide (3a)**. The solid residue was dried and crystallized from CH_3_OH (1 : 2) to yield 3a as yellow crystals (1.5 g, 75%). Mp 244 – 246.6°C. IR (KBr) (υ_max_, cm^–1^): 3181 (O-H) 3003 (N-H), 1682 (C=O), 1612 (C=N), 1567 and 1489(C=C), 1289(C-N), 1157(N-N)._1_H NMR (CDCl_3_, Me_4_Si) :^δ^H 7.1 and 7.28(5H, 2q, ^3^J_HH_= 7.37, arom), 8.16(1H, s, NCH), 8.06 and 9.33(4H, 2d, ^3^J_HH_= 5.28, pyridine), 11.04(1H, d, OH), 12.99(1H, s, NH).^13^C NMR (CDCl_3_, Me_4_Si) :^δ^C 117.14, 119.65, 128.24, 130.62, and 155.74 (arom), 126.05, 138.92, and 148.79 (pyridine), 151.2(C_11_), 165.28(OCN). Anal. calcd for C_13_H_11_N_3_O_2_(241.26) : C, 64.66; H, 4.55; N, 17.40%. Found: C, 64.98; H, 4.87; N, 17.73%.

**N^4^-[(E)-2-Phenylethylidene] Isonicotinohydrazide (3b)**. White crystals(1.76 g, 88%). Mp 184 – 187°C. IR (KBr) (υ_max_, cm^–1^) : 3198 (N-H), 1700 (C=O), 1600 (C=N), 1489 and 1447(C=C), 1283(C-N), 1148(N-N). 1H NMR (CDCl_3_, Me_4_Si) :^δ^H 3.62(2H, d, ^3^J_HH_= 6.64Hz, CHCH_2_), 660(1H, t, tNCH), 6.72 and 7.13 (5H,2q, ^3^JHH = 7.05 Hz, arom), 8.46 and 9.21(4H,2d, ^3^J_HH_= 5.28, pyridine), 9.09(1H, s, NH).

^13^C NMR(CDCl_3_, Me_4_Si):^δ^C 34.31(C_12_), 126.9, 127.23, 127.43, and 136.33 (arom), 122.82, 143.02, 151.06 (pyridine), 154.87(NCH), 165.44(OCN). Anal. calcd for C_13_H_11_N_3_O (225.27) : C, 69.25; H, 4.88; N, 18.64%. Found: C, 69.63; H, 5.11; N, 18.92%.

**N ^4^ [(E,2E)-3-Phenyl-2-propenylidene] Isonicotinohydrazide (3c)**. Yellow crystals (1.84 g, 92%). Mp 200 – 203.3°C. IR (KBr) (υ_max_, cm^–1^) : 3233 (N-H), 1676(C=O), 1640 (C=N), 1451(C=C), 1308(C-N), 1174(N-N).^1^H NMR (CDCl_3_, Me_4_Si) :^δ^H 6.06(1H, t, CHC), 6.94(1H, d, NCH), 7.51(1H, q, ^3^J_HH_= 15.97, CHCH), 7.25 and 7.44(5H, 2q, ^3^J_HH_= 2.12, 5.28, arom), 8.50 and 9.21(4H, 2d, ^3^J_HH_=7.83, pyridine).^13^C NMR (CDCl_3_, Me_4_Si) :^δ^C 124.76(C_13_), 127, 128.92, 129.14, and 135.81(arom), 130.42(C_12_), 144.62(C_11_), 122.83, 143.55 and 151.06(pyridine), 165.05(OCN). Anal. calcd for C_15_H_13_N_3_O (251.30): C, 62.70; H, 5.17; N, 16.71%. Found: C, 63.02; H, 5.34; N, 17.07%.

**N^4^[(E)ethylidene] Isonicotinohydrazide (3d)**. White crystals (1.3 g, 65%). Mp 244 – 246.6°C. IR (KBr) (υ_max_, cm^–1^): 3026 (N-H), 1660 (C=O), 1600 (C=N), 1410(C=C), 1300(C-N), 1164(N-N).^1^H NMR (CDCl_3_, Me_4_Si): δ_H_7.55 and 6.52(2H, 2s, ^2^J_HH_= 8.45, NCH), 8.45 and 9.21(4H, 2d, ^3^J_HH_= 5.28, pyridine), 9.25(1H, s, NH).^13^C NMR (CDCl_3_, Me_4_Si) :^δ^C 122.82, 143.6, and 151.06 (pyridine), 136.21(C_11_), 165.6(OCN). Anal. calcd for C_7_H_7_N_3_O (149.14): C, 56.32; H, 4.69; N, 28.16%. Found: C, 56.65; H, 4.93; N, 28.43%.

### *In vitro* activities

The compounds were assayed for cytotoxicity *in vitro* against K562 (human chronic myeloid leukemia) cells and Jurkat (human T lymphocyte carcinoma) cells.

The two cell lines were provided by the *Pasteur Institute* Laboratory of Natural and Biomimetic in Iran. The procedure for cytotoxicity studies was similar to that reported earlier.[9] Briefly, in order to calculate the concentration of each drug that produced a 50% inhibition of cell growth (IC_50_), 190 mL of cell suspension (5 × 10^4^cell/mL) was exposed to various concentrations of compounds dissolved in sterile Ethanol. The final concentration of Ethanol in the growth medium was 2% (v/v) or lower; the concentrations were without effect on cell replication.[10,11] After incubation periods of 72 hours for all cell lines, the cell concentrations were determined both in the control and in drug-treated cultures. All experiments were carried out six times and six series.

## RESULTS AND DISCUSSION

### Preparation of NHPM, NPI, NPPI, and NEI compounds

The reaction of aldehydes with 4-pyridinecarboxylic acid hydrazide, results in the formation of Isonicotinohydrazide compounds by the water eliminated mechanism. These compounds are quite stable and can be stored without any appreciable change. These are insoluble in common organic solvents, such as, dichloromethane, chloroform, hexane, and benzene. However, they are soluble in ethanol, THF, DMSO, and DMF. Their structures have been characterized by elemental analysis, ^1^H NMR, ^13^CNMR, and IR. Their elemental analyses are in agreement with their proposed formula. The spectral data of the compounds have a good relationship with the literature data.

NHPM, NPI, NPPI, and NEI compounds have been tested against two human cancer cell lines : K562 and Jurkat. The IC_50_cytotoxicity values of the compounds were compared with those found for starting organic bases as well as for some of the anti-cancer agents used nowadays.[10–12]

### Cytotoxicity studies

The general method used for testing on anti-tumor properties of these compounds is the standard testing method that has been previously described in greater detail: After 12 hours of pre-incubation at 37°C in 5% CO2 and 100% humidity atmosphere, the new compounds were added in the following concentration ranges:

0.1 – 610 µM for NHPM, 0.1 – 680 µM for NPI, 0.1 – 450 µM for NPPI, and 0.1 – 300 µM for NEI.

The compounds were first dissolved in ethanol and then filtrated. The incubation continued up to 72 hours and at the end of this period, the IC_50_for each compound was measured using Trypan blue. The IC_50_value was the compound concentration needed for killing 50% of the tumor cells that were determined by comparison of the number of tumor cells in the control plate (blank plate : the sample plate without compound) and test plate. The corresponding IC_50_and IC_90_(50 and 90% inhibitory dose) values are shown in Table 1.

**Table 1:** 72 hour IC_50_ values (µM) obtained for four compounds

Compound	IC_50_ for Cell line		IC_90_ for Cell line	
	K562	Jurkat	K562	Jurkat
NHPM	> 75	> 70	> 100	> 100
NPI	> 100	> 90	> 130	> 125
NPPI	> 90	> 80	> 115	> 110
NEI	> 100	> 85	> 110	> 110

## CONCLUSION

It is clear from the earlier discussion that NHPM, NPI, NPPI, and NEI compounds offer a new outlook for chemotherapy. The results of antitumor activity show that the compounds exhibit antitumor properties, and it is important to note that they show enhanced inhibitory activity. The mechanism by which these compounds act as antitumor agents is apoptosis. It has also been proposed that concentration plays a vital role in increasing the degree of inhabitation.[13–16]
